# Phytoplankton Diversity Effects on Community Biomass and Stability along Nutrient Gradients in a Eutrophic Lake

**DOI:** 10.3390/ijerph14010095

**Published:** 2017-01-20

**Authors:** Wang Tian, Huayong Zhang, Lei Zhao, Feifan Zhang, Hai Huang

**Affiliations:** Research Center for Engineering Ecology and Nonlinear Science, North China Electric Power University, Beijing 102206, China; tianwang870822@163.com (W.T.); zhaolei@ncepu.edu.cn (L.Z.); china907a@163.com (F.Z.); bjecology@gmail.com (H.H.)

**Keywords:** phytoplankton species richness, Shannon diversity index, community biomass, resource use efficiency, community turnover, spatial stability, nutrient gradients

## Abstract

The relationship between biodiversity and ecosystem functioning is a central issue in ecology, but how this relationship is affected by nutrient stress is still unknown. In this study, we analyzed the phytoplankton diversity effects on community biomass and stability along nutrient gradients in an artificial eutrophic lake. Four nutrient gradients, varying from slightly eutrophic to highly eutrophic states, were designed by adjusting the amount of polluted water that flowed into the lake. Mean phytoplankton biomass, species richness, and Shannon diversity index all showed significant differences among the four nutrient gradients. Phytoplankton community biomass was correlated with diversity (both species richness and Shannon diversity index), varying from positive to negative along the nutrient gradients. The influence of phytoplankton species richness on resource use efficiency (RUE) also changed from positive to negative along the nutrient gradients. However, the influence of phytoplankton Shannon diversity on RUE was not significant. Both phytoplankton species richness and Shannon diversity had a negative influence on community turnover (measured as community dissimilarity), i.e., a positive diversity–stability relationship. Furthermore, phytoplankton spatial stability decreased along the nutrient gradients in the lake. With increasing nutrient concentrations, the variability (standard deviation) of phytoplankton community biomass increased more rapidly than the average total biomass. Results in this study will be helpful in understanding the phytoplankton diversity effects on ecosystem functioning and how these effects are influenced by nutrient conditions in aquatic ecosystems.

## 1. Introduction

A high level of biodiversity loss is occurring due to global climate change and human activities [1]. The rapid loss of biodiversity has generated great concerns about the influence of diversity on important indicators of ecosystem function, such as productivity and stability [1,2,3,4]. Aquatic ecosystems are vulnerable and the loss of biodiversity may cause catastrophic consequences such as algae blooms or secondary extinctions [5,6]. Thus, studies on the relationship between phytoplankton diversity and ecosystem functioning are essential to develop appropriate conservation strategies in aquatic ecosystems [5,7].

Previously, studies related to the biodiversity effects on ecosystem functioning were mainly focused on terrestrial ecosystems, particularly grasslands [8,9]. A positive relationship between diversity and productivity was observed and supported by many laboratory and field experiments [8,10]. The potential underlying mechanisms of the diversity–productivity relationship include sampling effect and complementarity effect [11,12], i.e., a more diverse community has a higher probability to contain highly productive species (sampling effect), and can be complementary in the use of resources (complementarity effect). Aquatic ecosystems are special because their primary producers, mainly phytoplankton species, have a short generation time, efficient trophic transfer, and are sensitive to environmental factors [13,14,15]. Therefore, the diversity–productivity relationship derived from terrestrial ecosystems may not be directly applied to phytoplankton communities. Most studies based on artificial phytoplankton communities showed a positive relationship between diversity and productivity [7,16,17]. There have also been experimental studies that showed neutral, negative, or even more complex patterns between phytoplankton diversity and productivity [18,19,20]. In natural ecosystems, Zimmerman and Cardinale [21] reported that algal diversity had a positive influence on community biomass based on data from North American lakes. Vallina et al. [22] showed a unimodal relationship between the diversity and biomass of marine phytoplankton communities. Furthermore, ecologists have found that the effects of diversity on community biomass will vary with time and environment. Cardinale et al. [23] reported that the impacts of producer diversity on biomass production increased through time because of species complementarity. Weis et al. [19] suggested that the effects of algal diversity on primary production changed in a predictable sequence through successional time. Steudel et al. [24] found that the positive effects of microalgae biodiversity on biomass were decreased with increasing heat and salinity stress intensity. It is commonly known that nutrients play an important role in maintaining the growth rates, metabolic rates, and photosynthesis of phytoplankton communities [25]. However, whether phytoplankton diversity effects on community biomass are influenced by nutrient concentrations remains unknown.

Phytoplankton resource use efficiency (RUE) is an important aspect of ecosystem functioning and reflects the ability of phytoplankton to capture limiting resources [26,27,28]. It is commonly believed that phytoplankton species richness would benefit RUE at both regional and ecosystem scales [26,27,29]. However, Filstrup et al. [28] observed negative effects of phytoplankton diversity on RUE in heavy eutrophic lakes where the phytoplankton communities were mostly dominated by few cyanobacteria genera. Therefore, there is no consistent relationship between phytoplankton diversity and RUE, and this relationship may be influenced by nutrient conditions in a lake.

Ecosystem stability is another important aspect of ecosystem functioning; it contains a wide range of components, such as temporal stability, spatial stability, resistance, resilience, and others [30,31]. Ecologists have found that diversity would benefit the stability of an ecosystem by increasing the temporal mean and decreasing the temporal variance of biomass in a fluctuating environment [32]. The potential underlying mechanisms of the diversity–stability relationship include overyielding, portfolio effect, and species asynchrony [33,34]. Corcoran and Boeing [17] observed that phytoplankton stability was positively correlated with diversity. McGrady-Steed et al. [35] suggested that aquatic microbial communities with higher diversity were more stable (as measured by predictability). However, Gonzalez and Descamps-Julien [36] observed no relationship between the species richness and biomass stability of an artificial algae community. Moreover, recent field investigations showed conflicting diversity–stability relationships in phytoplankton communities [26,28]. Ptacnik et al. [26] discovered a negative relationship between phytoplankton taxonomic richness and community turnover. However, Filstrup et al. [28] reported the contrary conclusion that more diverse phytoplankton communities would generate higher community turnover in heavy eutrophic lakes. Therefore, the relationship between phytoplankton diversity and stability in natural ecosystems is complex and may be affected by trophic conditions.

In this research, four nutrient gradients (varying from slightly eutrophic to highly eutrophic states) were designed in an artificial eutrophic lake (Lake Qixinghu, Zaozhuang, China) by adjusting the amount of polluted water that flowed into the lake. The phytoplankton communities and main environmental factors under different nutrient gradients were investigated and measured. The effects of phytoplankton diversity (measured as species richness and Shannon diversity index) on community biomass, RUE, and community turnover under different nutrient gradients were analyzed. The spatial stability of phytoplankton community under different nutrient concentrations was also analyzed. The purpose of the present study was to explore whether phytoplankton diversity effects on ecosystem functioning were influenced by nutrient conditions.

## 2. Materials and Methods

### 2.1. Study Area

**Figure 1:**
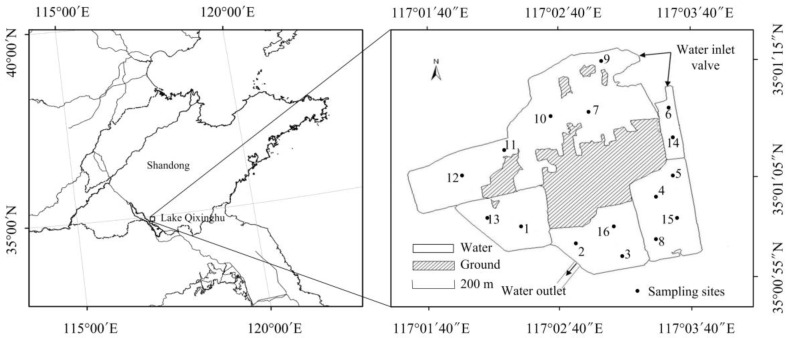
Location of Lake Qixinghu and the sample sites. This figure was made by ArcGIS version 10.0 (ESRI, Redlands, CA, USA).

Lake Qixinghu is located in the Shandong Province in China (Figure 1). It is an artificial lake at the base of a coal mine subsidence area. The total surface area of the lake is 2.05 km^2^, with a mean depth of about 1.5 m and a capacity of 2.16 × 10^6^ m^3^. The climate of the area is warm temperate monsoon with an annual average temperature of 13.7 °C [37]. The annual average rainfall is 550 mm to 720 mm, and nearly 60% of the precipitation occurs during the rainy summer [37]. Water from River Chenghe, a small river nearby the lake, can flow into Lake Qixinghu. The lake has a manual operation water inlet valve, which can help to control the amount of water that flows into the lake. River Chenghe contains high concentrations of nitrogen and phosphorus, since it is the fosse (moat, a small river built around a city in ancient times to protect the city from attack) of Zaozhuang and a large amount of industrial wastewater and agricultural runoff flow into the river. Therefore, the manual operation water inlet valve can control the nutrient conditions in the lake.

### 2.2. Experimental Design

At the beginning of the experiment, the manual operation water inlet valve was closed and the lake had no external pollution. Sampling was conducted at the beginning of June 2012. This situation provided low nutrient concentrations in the lake and was labeled as Scenario 1. We opened the manual operation water inlet valve to a relatively low level (about 1.0 × 10^4^ m^3^/day). After 30 days, sampling was conducted at the beginning of July. This situation provided relatively high nutrient concentrations in the lake and was labeled as Scenario 2. After the second investigation, we opened the manual operation water inlet valve to a relatively high level (about 2.0 × 10^4^ m^3^/day). After 30 days, sampling was conducted at the beginning of August. This situation provided high nutrient concentrations in the lake and was labeled as Scenario 3. We opened the manual operation water inlet valve to a high level (about 4.0 × 10^4^ m^3^/day). After 30 days, sampling was conducted at the beginning of September. This situation provided very high nutrient concentrations in the lake and was labeled as Scenario 4. The amount of polluted water that flowed into the lake per day was estimated by measuring the mean water depth, mean width of the water inlet, and the water velocities. Water velocities were measured using a SonTek 16 MHz 3D Micro Acoustic Doppler Velocimeters (SonTek, San Diego, CA, USA).

### 2.3. The Trophic States of the Lake

The trophic states of the lake in different scenarios were determined by comprehensive nutritional status index using the mean values of total nitrogen (TN), total phosphorus (TP), and water transparency (SD) [38]. The index was calculated by the following equation:

(1) $$TLI=\sum_{j=1}^{m}W_{j}TLI(j)$$

here TLI is the trophic state index of a lake; TLI(j) is the index of environmental variable j; W_j_ is the weight of environmental variable j. TLI(j) was calculated by the following equations:

(2) TLI(TN)=10(5.453+1.694ln(TN))

(3) TLI(TP)=10(9.436+1.624ln(TP))

(4) TLI(SD)=10(5.118-1.94ln(SD))

The weights of the three variables were calculated by the relative weight of their correlation coefficients with chlorophyll *a* in China lakes using the following equation:

(5) $$W_{j}={r_{j}}^{2}/\sum_{i=1}^{3}{r_{j}}^{2}$$

here *r_j_* is the correlation coefficient of TN, TP, or SD with chlorophyll *a* in China lakes, and their values were 0.82, 0.84, and −0.83, respectively [38]. Therefore, the weights of the three variables were 0.325, 0.342, and 0.333, respectively. Trophic states of a lake are set as follows: oligotrophic, TLI ≤ 30; mesotrophic, 30 < TLI ≤ 50; slightly eutrophic, 50 < TLI ≤ 60; moderately eutrophic, 60 < TLI ≤ 70; highly eutrophic, TLI > 70 [38].

### 2.4. Sampling and Measurements

A total of 16 sampling sites were uniformly set according to the distribution of the lake (Figure 1). Water temperature (WT), pH, and dissolved oxygen (DO) were measured in situ using YSI Professional Plus (YSI Incorporated, Yellow Springs, OH, USA) in the lake. The values of water transparency (SD) were measured using a Secchi disk. Water quality samples were collected using a Tygon tube water sampler at 0.50 m under the water surface. The samples were stored in acid-cleaned glass bottles at 4 °C and filtered through a 0.45-μm acetate filter for subsequent analyses. The concentration of total nitrogen (TN) was measured using the potassium persulfate oxidation-UV spectrophotometry method [39], and total phosphorus (TP) was determined using the Mo-Sb Anti-spectrophotometry method [40].

Phytoplankton samples (1 L) were collected under the water surface (0.5 m) at each sampling site and subsequently preserved in acidified Lugol’s solution for 24 h and condensed to 30 mL. A 0.1 mL aliquot of the condensed sample was added to a phytoplankton counting box to identify the species and count the cells of each species. The biomass of phytoplankton species was calculated based on the cell volume of each species [41]. When analyzing the phytoplankton diversity-productivity relationships in field lakes, ecologists generally used the standing stock biomass measured at a single time [21,42,43]. Therefore, we explored the relationship between phytoplankton diversity and community biomass in the present research. Phytoplankton species richness was determined by the number of species identified at each site. Phytoplankton Shannon diversity index was calculated using the following equation [44]:

(6) $$H=-\sum_{i=1}^{S}P_{i}\log P_{i}$$

here *P_i_* is the biomass proportion of species *i* in each sample and *S* is the species richness. *H* is the Shannon diversity index.

### 2.5. Calculation of Phytoplankton RUE and Stability

Phytoplankton RUE was measured as the biomass of phytoplankton per unit of limiting resources. Previous studies have usually used TN or TP as the limiting resources of phytoplankton [26,28]. In most sites of Lake Qixinghu, the ratios of TN:TP were below the Redfield value (planktonic biomass contains nitrogen and phosphorus in an atomic ratio of 16:1) [45]. In addition, TN had a high correlation with phytoplankton biomass (linear Pearson correlation: *R* = 0.591, *p* < 0.001, *N* = 64). Therefore, phytoplankton RUE was calculated as phytoplankton biomass per unit TN in this research.

We used community turnover to represent the temporal stability of phytoplankton, and it was calculated by Bray-Curtis dissimilarity between two samples using the following equation [26,28]:

(7) $$BC=\frac{\sum_{i=1}^{n}\left|y_{i1}-y_{i2}\right|}{\sum_{i=1}^{n}\left(y_{i1}+y_{i2}\right)}$$

here *BC* was the Bray-Curtis dissimilarity index between samples 1 and 2; *y_i_*_1_ and *y_i_*_2_ were the biomasses of phytoplankton species *i* in the two samples; *n* was the total number of phytoplankton species that appeared in both samples. We calculated the community turnover between each of the two consecutive measurements in the lake. The value of *BC* ranged from 0 to 1, with high *BC* representing large community turnover (i.e., low community stability) [28]. The numerator of Equation (7) stands for the summed variation of biomass and the denominator represents the total biomass. The relationships between the two components (summed variation of biomass and total biomass) and phytoplankton diversity can help to determine whether diversity influences stability through increasing the temporal mean or decreasing the temporal variation of biomass.

The spatial stability indices (*TSI*) of different phytoplankton taxa were measured as the coefficient of variation (the variance of biomass related to the mean value in different sites). The index was calculated using the following equation [8,46,47]:

(8) $$TSI=\frac{\mu }{\sigma }=\frac{\sum Bio}{\sqrt{\sum Var+\sum Cov}}$$

here *TSI* is the spatial stability index of different phytoplankton taxa; *μ* is the average total biomass of each taxon; and *σ* is the standard deviation of the total biomass. The standard deviation included the summed variance of each species (*Var*) and summed covariance of each two species (*Cov*).

The Kolmogorov-Smirnov method was used to test whether the data of diversity (both species richness and Shannon diversity index) were normally distributed [48]. The differences of phytoplankton community in the four scenarios were compared by analysis of similarities (ANOSIM) based on the Bray-Curtis dissimilarity coefficient [49]. The differences of environmental factors, phytoplankton biomass, diversity, RUE, and community turnover among the four scenarios were analyzed using one-way analysis of variance (ANOVA). Prior to analysis, the Kolmogorov-Smirnov method was used to test whether the data were normally distributed, and the Bartlett test was performed to assess the homogeneity of variance of the data. Post hoc comparisons were applied using the Tukey honestly significant difference (HSD) test at a significance level of 0.05. The influence of phytoplankton diversity on community biomass, RUE, and community turnover was analyzed by linear regression. Since the calculation of *BC* needed two measurements for each sampling site, phytoplankton species richness and Shannon diversity index were expressed as the initial values of each site.

## 3. Results

### 3.1. Environmental Factors in Different Scenarios

WT in Scenario 1 ranged from 22.4 °C to 24.7 °C, with a mean value of 23.6 ± 0.64 °C (mean ± standard deviation) in the lake (Table 1). Average values of WT in Scenarios 2, 3, and 4 were 25.8 ± 1.25 °C, 24.6 ± 1.27 °C, and 24.2 ± 1.76 °C, respectively. There were significant differences of WT among the four scenarios (one way ANOVA: *F*_(3, 60)_ = 9.12, *p* < 0.001), with a mean value in Scenario 2 significantly higher than that in the other three scenarios (all *p* < 0.05 by post hoc Tukey HSD). The lake was in a weak alkaline state, and mean values of pH were 7.90 ± 0.44, 8.45 ± 1.43, 7.82 ± 0.77, and 8.39 ± 0.66, respectively, in the four scenarios (Table 1). There was no significant difference of pH among the four scenarios (one way ANOVA: *F*_(3, 60)_ = 2.07, *p* = 0.113). Mean DO concentrations gradually decreased along the four scenarios, and their values were 8.69 ± 1.45 mg/L, 6.41 ± 0.93 mg/L, 5.92 ± 1.32 mg/L, and 4.58 ± 2.15 mg/L, respectively. There were significant differences of DO among different scenarios (one way ANOVA: *F*_(3, 60)_ = 10.05, *p* < 0.001), with a mean value in Scenario 1 significantly higher than that of others (all *p* < 0.05 by post hoc Tukey HSD). The lake had a low SD and its value was lower than 1 m in all sites, as shown in Table 1. The mean value of SD in Scenario 1 was significantly higher than that in the other scenarios (all *p* < 0.001 by post hoc Tukey HSD after one way ANOVA). The nutrient concentrations in the lake were high, as shown in Table 1. The mean concentration of TN ranged from 1.03 ± 0.23 mg/L to 7.17 ± 1.52 mg/L across the different scenarios. There were significant differences of mean TN concentrations between each of the two scenarios (all *p* < 0.05 by post hoc Tukey HSD after one way ANOVA). Average values of TP ranged from 0.20 ± 0.03 mg/L to 0.87 ± 0.15 mg/L at the four scenarios. There were also apparent variations of mean TP concentrations between each of the two scenarios (all *p* < 0.05 by post hoc Tukey HSD after one way ANOVA). The trophic state indices of the four scenarios were 58.96, 68.18, 75.65, and 82.23, respectively. Therefore, the trophic state of the lake was slightly eutrophic in Scenario 1, moderately eutrophic in Scenario 2, and highly eutrophic in Scenarios 3 and 4.

### 3.2. Phytoplankton Community Compositions in Different Scenarios

A total of 78 phytoplankton species belonging to 49 genera and 7 phyla were identified in the lake. The phytoplankton community included 31 Chlorophyta species, 21 Bacillariophyta species, and 15 Cyanophyta species. Phytoplankton species richness ranged from 71 in Scenario 1 to 55 in Scenario 4. Phytoplankton species names in different scenarios are shown in Table S1.

The phytoplankton biomass showed apparent variations in different scenarios. In Scenario 1, it ranged from 1.63 mg/L to 12.35 mg/L with an average value of 5.34 mg/L. The mean biomasses of Bacillariophyta, Chlorophyta, and Cyanophyta were 1.89 mg/L, 1.35 mg/L, and 0.97 mg/L, respectively, which accounted for 35.4%, 25.3%, and 18.2%, respectively, of the total phytoplankton biomass. However, in Scenario 2, phytoplankton biomass varied between 2.04 mg/L and 18.92 mg/L with an average value of 7.32 mg/L. The mean biomasses of Bacillariophyta, Chlorophyta, and Cyanophyta accounted for 45.6%, 20.2%, and 20.1%, respectively, of the total phytoplankton biomass. In Scenario 3, mean phytoplankton biomass reached 17.22 mg/L and the mean values of Bacillariophyta, Chlorophyta, and Cyanophyta were 6.13 mg/L, 3.39 mg/L, and 4.52 mg/L, respectively. The biomass proportions of the three taxa were 35.6%, 19.7%, and 26.2%, respectively. In Scenario 4, the mean biomass of phytoplankton reached 23.7 mg/L. The biomass proportions of Bacillariophyta, Chlorophyta, and Cyanophyta accounted for 26.5%, 26.2%, and 31.3%, respectively, of the total phytoplankton biomass. The biomass proportion of Cyanophyta increased gradually with nutrient gradients in the lake.

Results of ANOSIM showed that there were significant differences of phytoplankton in the four scenarios (*R* global = 0.330, *p* < 0.001). However, the phytoplankton communities of Scenarios 1 and 2 did not differ from each other (*R* = 0.043, *p* = 0.130). There were significant variations of phytoplankton community among other scenarios (for Scenarios 1 and 3, *R* = 0.521, *p* < 0.001; for Scenarios 1 and 4, *R* = 0.581, *p* < 0.001; for Scenarios 2 and 3, *R* = 0.344, *p* < 0.001; for Scenarios 2 and 4, *R* = 0.442, *p* < 0.001; for Scenarios 3 and 4, *R* = 0.093, *p* = 0.018).

**Figure 2:**
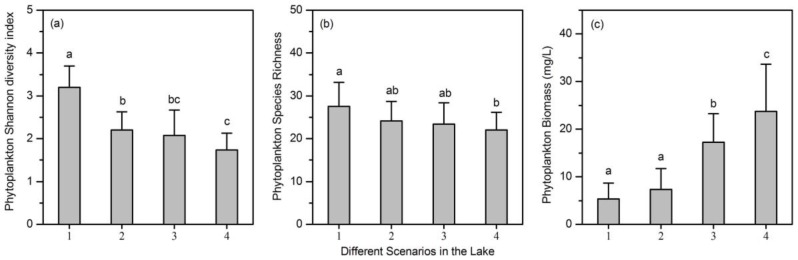
Mean values of (**a**) phytoplankton Shannon diversity index; (**b**) species richness; and (**c**) biomass in the four scenarios. Error bars are the standard deviations of the sample sites in Lake Qixinghu. Significant differences among the four scenarios are marked by letters (a, b, and c).

There were significant differences among the phytoplankton Shannon diversity index values in the four scenarios (one way ANOVA: *F*_(3, 60)_ = 27.2, *p* < 0.001), with a mean value in Scenario 1 significantly higher than that in the other scenarios (all *p* < 0.001 by post hoc Tukey HSD), as shown in Figure 2. Mean phytoplankton species richness in Scenario 1 was significantly higher than that in Scenario 4 (*p* < 0.05 by post hoc Tukey HSD after one way ANOVA). Thus, phytoplankton diversity (both species richness and Shannon diversity index values) significantly decreased along nutrient gradients in the lake (Figure 2). Phytoplankton biomass also showed significant differences among the four scenarios (one way ANOVA: *F*_(3, 60)_ = 28.8, *p* < 0.001), with mean values in Scenarios 3 and 4 significantly higher than those in Scenarios 1 and 2 (all *p* < 0.001 by post hoc Tukey HSD).

### 3.3. Relationship Between Phytoplankton Diversity and Biomass

**Figure 3:**
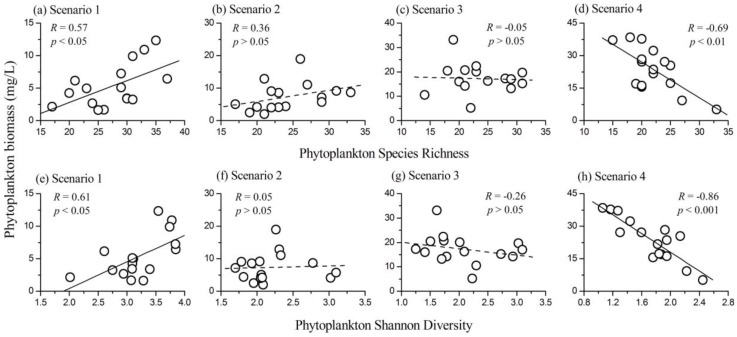
The relationships between phytoplankton species richness and community biomass in (**a**) Scenario 1; (**b**) Scenario 2; (**c**) Scenario 3; and (**d**) Scenario 4. The relationships between phytoplankton Shannon diversity index and community biomass in (**e**) Scenario 1; (**f**) Scenario 2; (**g**) Scenario 3; and (**h**) Scenario 4.

The diversity (both species richness and Shannon diversity index) of the four scenarios were all normally distributed (all *p* > 0.05 by Kolmogorov-Smirnov test). When the lake was in the slightly eutrophic state (Scenario 1), phytoplankton species richness was positively correlated with community biomass (*R* = 0.57, *p* < 0.05, *N* = 16), as shown in Figure 3a. However, there were no apparent relationships between phytoplankton species richness and biomass in Scenarios 2 and 3 (for Scenario 2, *R* = 0.36, *p* > 0.05, *N* = 16; for Scenario 3, *R* = −0.05, *p* > 0.05, *N* = 16). A strong negative relationship between the two variables was observed in Scenario 4 when the lake was in the highly eutrophic state (*R* = −0.69, *p* < 0.01, *N* = 16; Figure 3d). Phytoplankton Shannon diversity index also showed strong relationships with community biomass, varying gradually from positive in Scenario 1 (*R* = 0.61, *p* < 0.05, *N* = 16) to negative in Scenario 4 (*R* = −0.86, *p* < 0.001, *N* = 16). However, in Scenarios 2 and 3, phytoplankton Shannon diversity index had no significant influence on community biomass (for Scenario 2, *R* = 0.05, *p* > 0.05, *N* = 16; for Scenario 3, *R* = −0.26, *p* > 0.05, *N* = 16). Therefore, the relationship between phytoplankton diversity (both species richness and Shannon diversity index) and community biomass varied along nutrient gradients in the lake.

### 3.4. Relationship between Phytoplankton Diversity and RUE

**Figure 4:**
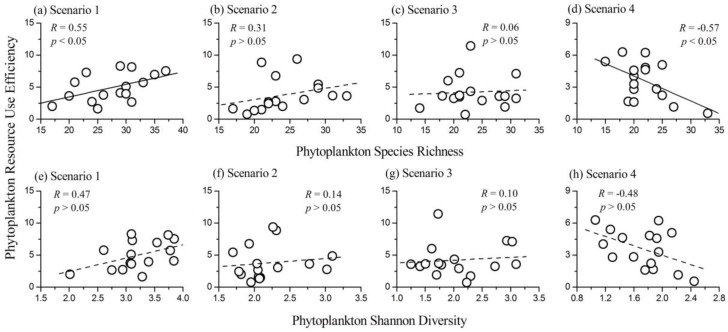
The relationships between phytoplankton species richness and resource use efficiency (RUE) in (**a**) Scenario 1; (**b**) Scenario 2; (**c**) Scenario 3; and (**d**) Scenario 4. The relationships between phytoplankton Shannon diversity index and RUE in (**e**) Scenario 1; (**f**) Scenario 2; (**g**) Scenario 3; and (**h**) Scenario 4.

The relationship between phytoplankton species richness and RUE was different in the four scenarios and varied gradually from positive in Scenario 1 (*R* = 0.55, *p* < 0.05, *N* = 16) to negative in Scenario 4 (*R* = −0.57, *p* < 0.05, *N* = 16). In Scenarios 2 and 3, the correlation coefficients between the two variables were not significant (for Scenario 2, *R* = 0.31, *p* > 0.05, *N* = 16; for Scenario 3, *R* = 0.06, *p* > 0.05, *N* = 16). The influence of phytoplankton Shannon diversity index on RUE was not significant in the four scenarios, as shown in Figure 4. Therefore, phytoplankton RUE was more sensitive to the variation of species richness than Shannon diversity index.

### 3.5. Relationship between Phytoplankton Diversity and Community Turnover

**Figure 5:**
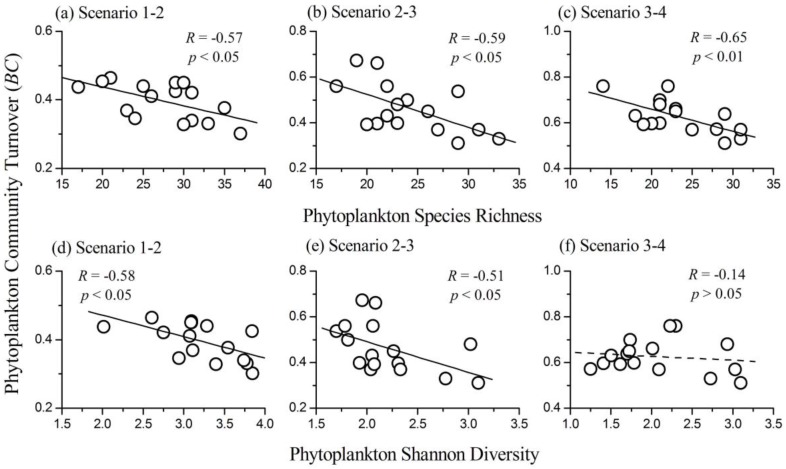
The relationships between phytoplankton species richness and community turnover in the situations of (**a**) Scenarios 1–2; (**b**) Scenarios 2–3; and (**c**) Scenarios 3–4. The relationships between phytoplankton Shannon diversity index and community turnover in the situations of (**d**) Scenarios 1–2; (**e**) Scenarios 2–3; and (**f**) Scenarios 3–4.

Mean values of phytoplankton community turnover were significantly increased along nutrient gradients in the lake (one way ANOVA: *F*_(2, 45)_ = 32.35, *p* < 0.001). Therefore, phytoplankton temporal stability decreased with increasing nutrient concentrations. Phytoplankton species richness was negatively correlated with community turnover (for Scenarios 1–2, *R* = −0.57; for Scenarios 2–3, *R* = −0.59; for Scenarios 3–4, *R* = −0.65; all *p* < 0.05, *N* = 16), which indicated a positive diversity–stability relationship in the lake (Figure 5). Further investigations showed that phytoplankton species richness had a positive relationship with total biomass (*R* = 0.49, *p* < 0.05, *N* = 16), but had no relationship with the summed variation of biomass (*R* = −0.27, *p* > 0.05, *N* = 16) when phytoplankton community changed from Scenario 1 to 2. Phytoplankton species richness was negatively correlated with both total biomass (*R* = −0.50, *p* < 0.05, *N* = 16) and the summed variation of biomass (*R* = −0.62, *p* < 0.05, *N* = 16) when phytoplankton community changed from Scenario 3 to 4. The influence of phytoplankton Shannon diversity on community turnover was similar to that of species richness for Scenarios 1–2 and 2–3 (for Scenarios 1–2, *R* = −0.58; for Scenarios 2–3, *R* = −0.51; all *p* < 0.05, *N* = 16). However, when phytoplankton community changed from Scenario 3 to 4, phytoplankton Shannon diversity had no significant influence on community turnover (*R* = −0.14, *p* > 0.05, *N* = 16). In summary, phytoplankton diversity had a negative influence on community turnover in most cases.

### 3.6. Phytoplankton Spatial Stability in Different Nutrient Gradients

**Figure 6:**
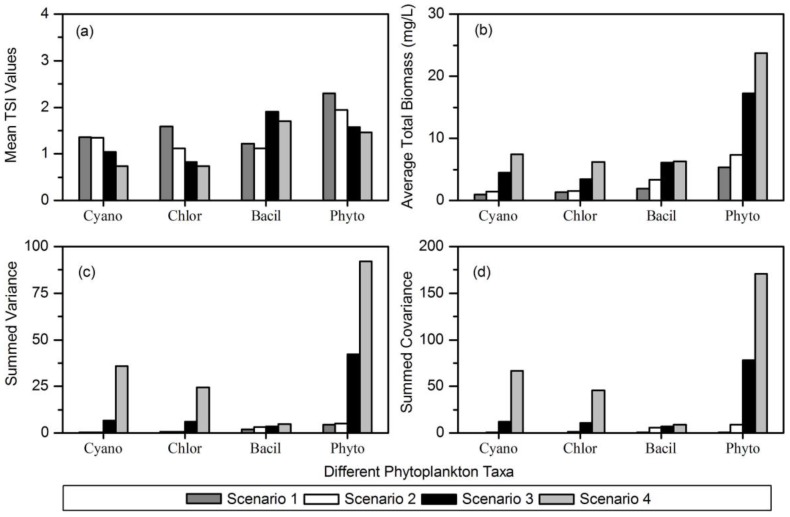
The values of (**a**) spatial stability index (*TSI*); (**b**) average total biomass; (**c**) summed variance; and (**d**) summed covariance of different phytoplankton taxa in the four scenarios. Cyano: Cyanophyta, Chlor: Chlorophyta, Bacil: Bacillariophyta, Phyto: total phytoplankton.

The spatial stability indices of Cyanophyta, Chlorophyta, and total phytoplankton decreased gradually from Scenario 1 to Scenario 4 (Figure 6a), supporting a negative relationship between spatial stability and nutrient concentrations. Further investigations showed that although the average total biomass of the three phytoplankton taxa increased with nutrient gradients (Figure 6b), the summed variance (Figure 6c) and covariance (Figure 6d) increased at a more rapid rate. However, the spatial stability indices of Bacillariophyta in Scenarios 3 and 4 were higher than those in Scenarios 1 and 2 (Figure 6a). The summed variance (Figure 6c) and covariance (Figure 6d) of Bacillariophyta showed little variation along nutrient gradients while the average total biomass increased with increasing nutrient concentrations (Figure 6b).

## 4. Discussion

Analyzing the influence of phytoplankton diversity on ecosystem functioning is crucial to understand the ecological consequences of diversity loss and develop appropriate conservation strategies in aquatic ecosystems. Here, we showed that phytoplankton diversity effects on both community biomass and RUE were influenced by nutrients, and varied gradually from positive to negative along the nutrient gradients (Figure 3). However, the relationship between phytoplankton diversity and community turnover was not influenced by nutrient gradients in the lake (Figure 5). Our results helped to quantify the effects of phytoplankton diversity on community biomass and stability, and how these effects changed along nutrient stress gradients.

When the lake was in the slightly eutrophic state (Scenario 1), phytoplankton community biomass had a positive relationship with both species richness and Shannon diversity index. Additionally, phytoplankton RUE had a strong positive relationship with species richness and a weak positive relationship with Shannon diversity index. Therefore, phytoplankton community with higher diversity was more efficient in capturing the limiting resources and led to a positive diversity–productivity relationship. This result was consistent with most diversity–productivity relationships obtained from both terrestrial [8,10] and aquatic ecosystems [7,16,17]. However, when the lake was in Scenario 4, phytoplankton diversity (both species richness and Shannon diversity index) was negatively correlated with community biomass. Results of one way ANOVA showed that both phytoplankton species richness and Shannon diversity index in Scenario 4 were significantly lower than those in the other scenarios. Phytoplankton communities with low diversity in highly eutrophic lakes are generally dominated by a few species (mainly Cyanophyta species). The increased Cyanophyta biomass proportion with increasing nutrient concentrations in Lake Qixinghu confirmed this conclusion. Cyanophyta species are more efficient in resource use than other phytoplankton taxa [28]. Thus, phytoplankton RUE had a strong negative relationship with species richness and a weak negative relationship with Shannon diversity index in Lake Qixinghu (Figure 4). Therefore, phytoplankton communities with lower diversity were much more efficient in resource use and led to a negative relationship between diversity and community biomass in the lake. Filstrup et al. [28] also reported a negative relationship between phytoplankton RUE and evenness in highly eutrophic lakes where the phytoplankton communities were mostly dominated by few Cyanobacteria genera. When Lake Qixinghu was in Scenarios 2 and 3, the influence of phytoplankton diversity (both species richness and Shannon diversity index) on both community biomass and RUE was not significant (Figure 3 and Figure 4). Combining the results of the present study and previous studies [7,16,17,28], we can conclude that the phytoplankton diversity effects on productivity were positive in oligotrophic, mesotrophic, and slightly eutrophic lakes, but were negative in highly eutrophic lakes.

We observed that phytoplankton community turnover had a strong negative correlation with diversity during each of the two consecutive scenarios (i.e., a positive relationship between diversity and temporal stability). Ecologists have found that diversity would benefit the stability of ecosystems by increasing the temporal mean and decreasing the temporal variance of biomass [32]. In this research study, phytoplankton diversity had a positive relationship with total biomass, but no relationship with summed variation of biomass when the phytoplankton community changed from Scenario 1 to 2. Thus, phytoplankton diversity stabilized the ecosystem mainly through increasing the temporal mean biomass when the lake was in the slightly eutrophic state. Phytoplankton diversity was negatively correlated with both the total biomass and summed variation of biomass when the phytoplankton community changed from Scenario 3 to 4. Therefore, phytoplankton diversity mainly influenced community stability by decreasing the temporal variation of biomass when the lake was in the highly eutrophic state. Our results confirmed that phytoplankton diversity had a positive influence on temporal stability at various nutrient conditions. However, the underlying mechanisms changed from increasing the temporal mean to decreasing the temporal variation of biomass with the increase of eutrophication. Results in this study were consistent with most previous studies [26,32,35]. However, Filstrup et al. [28] reported a negative relationship between phytoplankton diversity and community turnover. In the study of Filstrup et al. [28], the phytoplankton community was dominated by few Cyanophyta genera (the biomass proportions of Cyanophyta were higher than 75% at most sites) and had lost the ability to respond to environmental changes. Therefore, Cyanophyta may play an important role in influencing the stability of phytoplankton communities.

We also observed that the spatial stability of Cyanophyta, Chlorophyta, and total phytoplankton decreased with increasing eutrophication (Figure 6). Although the average total biomasses of the three taxa increased with nutrient gradients, the summed variance and covariance increased at a more rapid rate. Therefore, nutrient concentrations reduced the spatial stability of phytoplankton taxa by increasing the variability of biomass (standard deviation of biomass). However, the stability of Bacillariophyta increased with increasing nutrient concentrations. Eutrophication had a strong positive influence on the average total biomass, but had no influence on the summed variance and covariance of Bacillariophyta. Therefore, nutrient concentrations increased the spatial stability of Bacillariophyta by increasing the average total biomass. These results demonstrated that Bacillariophyta may be more stable in eutrophic lakes.

We observed that the relationship between phytoplankton species richness and RUE varied from positive to negative along the nutrient gradients in the lake. However, the influence of phytoplankton Shannon diversity index on RUE was not significant. Additionally, phytoplankton species richness and Shannon diversity index had different effects on community turnover in Scenario 4. Previously, ecologists showed that the influence of producer diversity on ecosystem functioning might be influenced by the metrics of diversity, especially in aquatic ecosystems [26,28,50]. Variations in phytoplankton species richness are often the result of species extinction or invasion, whereas changes of Shannon diversity index are generally related to variations in species dominance [50,51]. Results from our study showed that phytoplankton RUE and community turnover were more sensitive to variations in species richness than Shannon diversity index. On the basis of these results, phytoplankton species extinction or invasion in eutrophic lakes has great influence on ecosystem functioning. Additionally, a distinction should be made between species richness and Shannon diversity index when analyzing the relationship between phytoplankton diversity and ecosystem functioning.

In temperate regions, phytoplankton communities generally have apparent seasonal variations due to the fluctuations of environmental factors [52]. Tian et al. [47] showed that in Lake Nansihu (Jining, China), the largest freshwater lake in North China, phytoplankton biomass reached the maximum value in summer and decreased from July to September. In the present study, Lake Qixinghu has the same latitude and climate as Lake Nansihu. However, the phytoplankton biomass and Cyanophyta proportion continued to increase from June to September. Therefore, the variations of phytoplankton biomass in Lake Qixinghu were mainly influenced by the nutrient gradients, rather than seasonal variations.

The results of this study demonstrated the importance of phytoplankton diversity in maintaining ecosystem functioning and how the diversity effects were influenced by nutrient enrichment. Therefore, protecting species diversity and limiting anthropogenic import of nutrients are critical to prevent ecological catastrophe in eutrophic lakes.

## 5. Conclusions

In this study, we analyzed the effects of phytoplankton diversity on community biomass, RUE, and community turnover under different nutrient gradients in Lake Qixinghu. The main findings of the present research can be summarized as follows:

(1) A total of 78 phytoplankton species were identified in the lake. Phytoplankton biomass, species richness, and Shannon diversity index all showed significant differences among the nutrient gradients.
(2) The relationship between phytoplankton diversity (both species richness and Shannon diversity index) and community biomass changed from positive to negative along the nutrient gradients.
(3) The influence of phytoplankton species richness on RUE changed from positive to negative along the nutrient gradients. However, the influence of phytoplankton Shannon diversity index on RUE was not significant.
(4) Phytoplankton diversity (both species richness and Shannon diversity index) had a negative influence on community turnover in most cases (i.e., a positive relationship between diversity and temporal stability). The underlying mechanisms changed from increasing the temporal mean to decreasing the temporal variation of biomass along the nutrient gradients.
(5) The spatial stability of Cyanophyta, Chlorophyta, and total phytoplankton decreased with increasing nutrient concentrations in the lake. Eutrophication decreased the phytoplankton spatial stability mainly by increasing the temporal variance of community biomass.

The results in this study will be helpful in understanding the effects of phytoplankton diversity on ecosystem functioning, and how these effects are influenced by nutrient conditions.

## Figures and Tables

**Table 1:** Main environmental factors of the four scenarios.

Scenario	WT (°C)	pH	DO (mg/L)	SD (cm)	TN (mg/L)	TP (mg/L)
Scenario 1	23.6 ± 0.64 ^a^ (22.4, 24.7)	7.90 ± 0.44 ^a^ (7.22, 8.50)	8.69 ± 1.45 ^a^ (5.98, 10.51)	73.1 ± 11.2 ^a^ (58.0, 94.0)	1.03 ± 0.23 ^a^ (0.44, 1.91)	0.20 ± 0.03 ^a^ (0.12, 0.28)
Scenario 2	25.8 ± 1.25 ^b^ (23.9, 29.1)	8.45 ± 1.43 ^a^ (5.49, 10.93)	6.41 ± 0.93 ^b^ (5.17, 8.28)	42.2 ± 13.3 ^b^ (28.0, 70.0)	2.17 ± 0.48 ^b^ (1.18, 3.70)	0.30 ± 0.05 ^b^ (0.10, 0.67)
Scenario 3	24.6 ± 1.27 ^a^ (22.7, 26.2)	7.82 ± 0.77 ^a^ (6.17, 9.34)	5.92 ± 1.32 ^b^ (1.18, 11.3)	45.1 ± 13.5 ^b^ (30.0, 80.0)	4.84 ± 0.86 ^c^ (1.96, 7.46)	0.47 ± 0.09 ^c^ (0.13, 1.19)
Scenario 4	24.2 ± 1.76 ^a^ (21.7, 28.2)	8.39 ± 0.66 ^a^ (7.00, 9.32)	4.58 ± 2.15 ^b^ (1.10, 9.93)	45.2 ± 14.5 ^b^ (29.4, 76.0)	7.17 ± 1.52 ^d^ (2.77, 10.3)	0.87 ± 0.15 ^d^ (0.23, 2.05)

Values are expressed as mean ± standard deviations (minimum, maximum). WT: water temperature; DO: dissolved oxygen; SD: water transparency; TN: total nitrogen; TP: total phosphorus. Significant differences among the four scenarios are marked by letters (a, b, c, and d).

## Supplementary Materials

The following are available online at www.mdpi.com/1660-4601/14/1/95/s1, Table S1: The list of phytoplankton species in Lake Qixinghu.
